# Disparities in Healthcare: Evaluation of Equity in Access to Surveillance Colonoscopy After Hemicolectomy in Patients With Colon Cancer During the COVID-19 Pandemic

**DOI:** 10.7759/cureus.21582

**Published:** 2022-01-25

**Authors:** Ranbir Singh, Eshan Patel

**Affiliations:** 1 Internal Medicine, NewYork-Presbyterian Brooklyn Methodist Hospital, Brooklyn, USA; 2 Hematology/Oncology, Robert Wood Johnson Barnabas Health, Somerville, USA

**Keywords:** gi oncology, gastroenterology, hemicolectomy, colon cancer, colonoscopy surveillance

## Abstract

Introduction: Surveillance colonoscopy is rcommended for patients with colon cancer who obtain a hemicolectomy for tumor resection. Guidelines from many organizations require this colonoscopy to be performed within one year after resection. The objective of this study was to evaluate the difference in surveillance colonoscopy rates between white people and African Americans who had their colon tumors resected. The second objective was to determine whether the COVID-19 pandemic affected these colonoscopy rates. The study goal was to shed light on the issue of low colonoscopy rates among African Americans with colon cancer after tumor removal by hemicolectomy and on how the pandemic exacerbated this issue.

Methods: A total of 800 patient charts from Brooklyn Methodist Hospital were reviewed. The selected patients had a history of colon cancer and received hemicolectomy in the past. The patients were divided according to race and their expected surveillance colonoscopy dates. One group included patients with an expected one-year follow-up date for colonoscopy after hemicolectomy before the start of the pandemic. Another group included patients with colonoscopies due to be performed during the pandemic. A two-sample proportions test was used to compare the colonoscopy rates before and during the pandemic for African Americans. The two-sample equal variance t-test was used to compare the average distance from the patients' home to hospital between African Americans and whites.

Results: The surveillance colonoscopy rates among African Americans were 54% before and 45% during the pandemic. This difference was significant (p < 0.001). The colonoscopy rates between whites and African Americans differed. The surveillance colonoscopy rates among whites were 97% before and 84% during the pandemic. The distance between the patients' homes and the hospital where the procedure was performed also significantly differed. The average travel distance for whites was 1.33 miles and that for African Americans was 3.98 miles (p < 0.001). A total of 215 of the 416 African American patients included had tumors in the cecum and ascending colon.

Conclusion: A significant difference was observed in the colonoscopy rates for African Americans before and during the pandemic. A substantial difference was found in the colonoscopy rates between whites and African Americans, which increased during the pandemic. The distance from the patients' home to the hospital performing the colonoscopy was twice as far for African Americans than whites in the borough of Brooklyn. These data support the hypothesis that a significant difference in colonoscopy rates exists between African Americans and whites, probably because of a healthcare disparity in access to this procedure. The study objective was to highlight the long-standing issue of low colonoscopy rates in African Americans and how the pandemic further decreased these low rates.

## Introduction

Colonoscopy is an essential tool for colon cancer screening as part of patients' health maintenance. The most recent guidelines for colorectal cancer screening recommend colonoscopies at the age of 45 [1]; therefore, colonoscopies are a valuable resource. The rate of colon cancer is 20% higher among African Americans than whites in America [2]. This disparity in colon cancer incidence between these groups can be diminished with routine colonoscopies; however, the rate of screening in African Americans is lower than that in whites. Approximately, 48.9% of African Americans and 56% of whites receive colonoscopy screenings [3].

Approximately, 80% of colon cancers localize to the colon wall or regional nodes, and surgical resection is the only curative treatment [4]. Most guidelines suggest surveillance colonoscopy one year after hemicolectomy with anastomosis [5-7] because 90% of anastomosis recurrences are detected within three years after the primary resection [8].

Given that the rate of screening colonoscopies for healthy African American adults is less than 50% [3], the first objective of this study was to determine whether the rates for one-year surveillance colonoscopies after hemicolectomy among African American patients with colon cancer are also less than 50% and whether this rate further decreased during the COVID-19 pandemic. The COVID-19 pandemic has placed an immense strain on healthcare resources, including screening and essential endoscopic activity, thus resulting in a buildup of referrals and the postponement of colorectal cancer screening [9]. As the screening colonoscopy rates for African Americans are lower than those for whites, the second study objective was to determine whether one-year surveillance colonoscopy rates in patients with colon cancer after hemicolectomy are lower among African Americans than whites.

## Materials and methods

The project received New York Methodist Hospital IRB approval (1739382-1). Patients who had a history of colon cancer with hemicolectomy were included in this study. These patients were also chart reviewed for age, sex, race, the location of colon cancer, distance from the hospital, and data about whether they obtained surveillance colonoscopy within one year after resection. Patients who did not have colon cancer or hemicolectomy were excluded from the study.

A total of 800 patients were chart reviewed; 416 of the charts were from African American patients, and 384 were from white patients. These patients were further divided according to their expected surveillance colonoscopy dates. Patients who needed a colonoscopy before the COVID-19 pandemic were placed in one group and patients who needed a colonoscopy during the pandemic were placed in another group. The colonoscopy rates for each race before and during the pandemic were calculated. The two-sample proportions test compared the colonoscopy rates between African Americans before and during the pandemic. Bar graphs illustrated the difference in colonoscopy rates between African Americans and whites during these two timeframes. During data collection, no patients were contacted as a chart review was sufficient.

## Results

Colonoscopy rates between African Americans requiring surveillance colonoscopy before and during the pandemic were compared with a two-sample proportions test. The Z test was out of range of the Z critical upper and lower limits (Table 1). The p-value was <0.001; therefore, the null hypothesis of no significant difference in the colonoscopy rates between African Americans before and during the pandemic was rejected. The alternative hypothesis of a significant difference in rates between groups from these two timeframes was accepted.

**Table 1:** One-year surveillance colonoscopy rates status after colon tumor resection among African Americans before and during the COVID-19 pandemic

Statistics	Values
N (pre-pandemic)	212
N (pandemic)	204
Colonoscopy rates (pre-pandemic)	0.54
Colonoscopy rates (pandemic)	0.45
Z test	22.85
Z critical value lower limit	-1.96
Z critical value upper limit	1.96
p-value	<0.001

**Figure 1:**
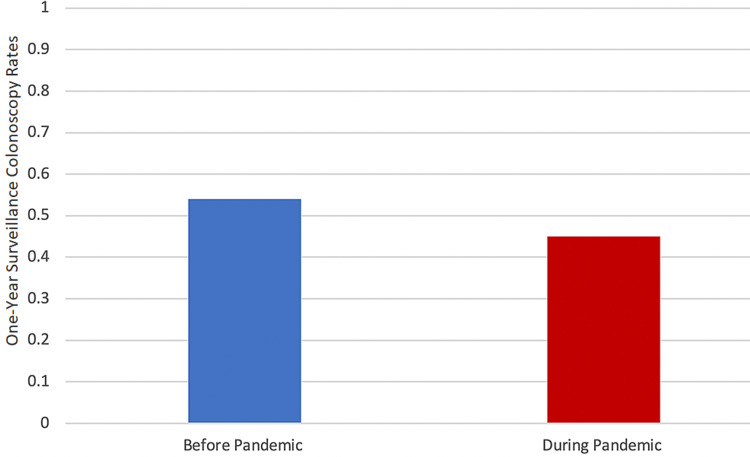
One-year surveillance colonoscopy rates status post colon tumor resection among African Americans before and during the COVID-19 pandemic

Figure 1 illustrates the difference in colonoscopy rates before and during the pandemic between African Americans: 54% obtained colonoscopies before the pandemic, and 45% received colonoscopies after the pandemic.

**Figure 2:**
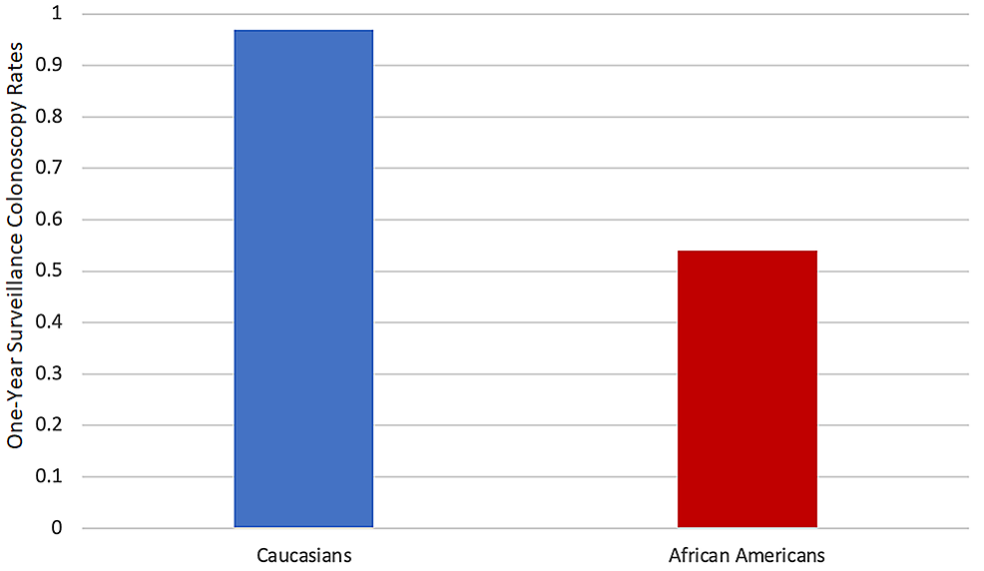
One-year surveillance colonoscopy status post colon tumor resection rates before the COVID-19 pandemic

Figure 2 compares the colonoscopy rates between whites and African Americans before the pandemic. The rate for whites was 97%, whereas the rate for African Americans was 54%.

**Figure 3:**
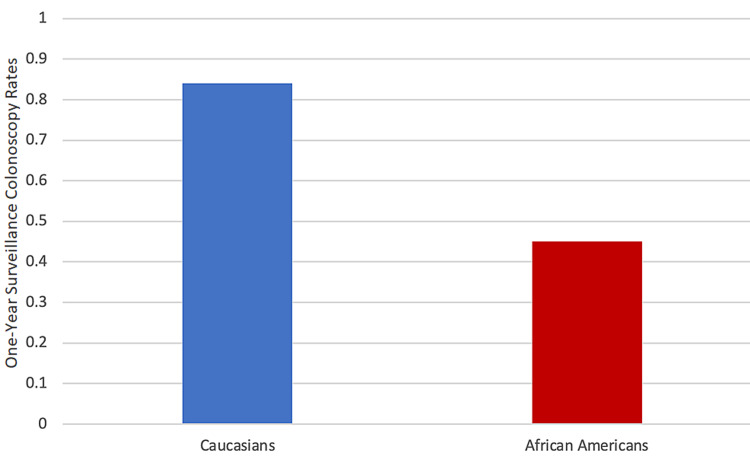
One-year surveillance colonoscopy status post colon tumor resection rates during the COVID-19 pandemic

Figure 3 compares the colonoscopy rates between whites and African Americans during the pandemic. The rate for whites was 84% and that for African Americans was 45%.

The average distance from the patients’ home to the hospital performing the colonoscopy was compared between whites and African Americans (Table 2). On average, whites had to travel 1.33 miles to their colonoscopy site, whereas African Americans had to travel 3.98 miles. The two-sample equal variance t-test was used to compare the average differences, and the average distances between the groups significantly differed (p < 0.001).

**Table 2:** Average distance to the hospital from the patient home for surveillance colonoscopies

Race	Distance (in miles)
African American	3.98
White	1.33
Maximum distance from the hospital (African American)	12.2
Maximum distance from the hospital (white)	5.2
P-value (two-sample equal variance T-test)	<0.001

**Figure 4:**
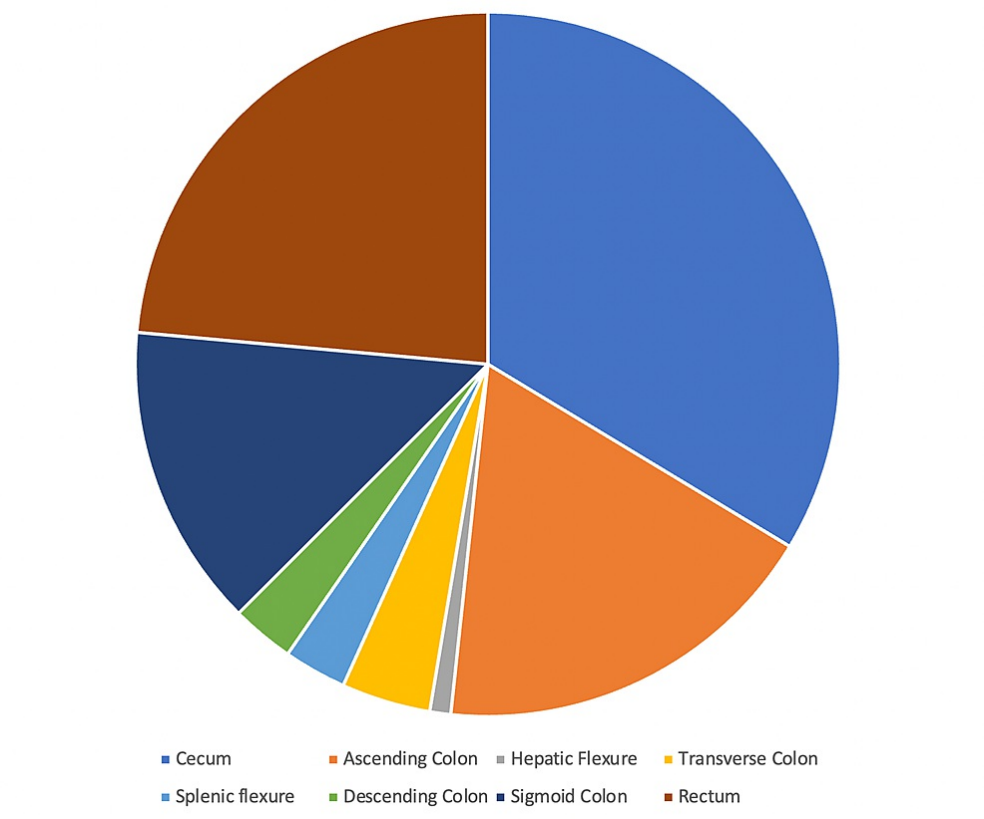
Colon tumor locations in African Americans

The colon tumor sites in African Americans are displayed in Figure 4. The most common tumor sites were the cecum and ascending colon, which accounted for 215 of the 416 charts reviewed for African American patients.

## Discussion

Colonoscopies are crucial for routine colon cancer screening. They are also an important resource for patients who have received colonic tumor resection. The five-year recurrence rate for stage I colon cancer is 10%, and this rate increases to 36% in patients with later disease stages [10]. As previously described, multiple guidelines, such as those from the American Society of Clinical Oncology and the US Multi-society Task Force, suggest that surveillance colonoscopy should be performed at least one year after tumor resection [5-7]. These guidelines indicate the importance of colonoscopies. The objective of this study was to determine whether colonoscopy resources are distributed equally, particularly during the COVID-19 pandemic.

The colonoscopy rates significantly decreased in African Americans during the pandemic (45% after and 54% before the pandemic, p < 0.001; Table 1 and Figure 1). The results also showed a disparity between whites and African Americans in the rates of post surveillance colonoscopies: 97% of whites received colonoscopies compared with 54% of African Americans (Figure 2). The disparity worsened during the COVID-19 pandemic: 84% of whites and only 45% of African Americans received surveillance colonoscopies (Figure 3). Although the colonoscopy rates decreased in both groups during the pandemic, less than 50% of African Americans received medically indicated colonoscopies, whereas more than 75% of whites received colonoscopies in the study. The data support the hypothesis that the colonoscopy rates between these races are not equal in the borough of Brooklyn, and the disparity worsened during the pandemic.

One possible cause of these unequal rates is disparities in colonoscopy access. The average distance that African Americans had to travel from their homes to the hospital was 3.98 miles with a maximum distance of 12.2 miles - distances nearly twice those for whites who had to travel an average of 1.33 miles and a maximum distance of 5.2 miles (Table 2). A study by Sly et al. that was aimed to identify barriers to colonoscopy screening among African Americans who do not adhere to the screening guidelines has reported that of the 100 African American patients interviewed, most had a lack of information and understanding regarding colorectal cancer. Another reason for avoiding screening was fear and anxiety, which caused most patients to either delay or cancel their colonoscopies altogether [3]. These factors are likely to contribute to the lower colonoscopy rates.

During the pandemic, colonoscopies were a complex procedure to perform in gastrointestinal clinical settings. In patients with COVID-19, colonoscopy places health professionals at a high risk of exposure to SARS-CoV-2 RNA in patients' stool samples [11]. This risk limited the number of colonoscopies that could be performed in one day, thus constraining the availability of post-resection surveillance colonoscopies. African Americans, in addition to having to travel twice the distance to the hospital than whites, also experience a lack of information and face fears/anxieties about colonoscopy. The restrictions caused by the COVID-19 pandemic probably exacerbated the already unequal colonoscopy rates between whites and African Americans. This finding is especially troubling because African Americans are at a higher risk of developing colorectal cancer than other racial groups [2].

The most common tumor sites in all African Americans in the study were the cecum and ascending colon: 215 of the 416 charts reviewed had a primary tumor in one of these regions (Table 2). A retrospective study by Jung et al., involving 974 patients who had undergone curative resection after being diagnosed with colon cancer, found that patients with right colon cancer, in the region of the colon proximal to the splenic flexure, have a higher colon cancer recurrence rate than patients with left colon cancer, in the region distal to the splenic flexure [12]. Due to these factors, surveillance colonoscopies after colonic tumor resection are ever more urgently needed for African Americans.

Limitations of this study include electronic medical records (EMR) access because the included patients might potentially have gone to another hospital to complete their surveillance colonoscopies, and the investigating team might not have access to that hospital's EMRs. Only 800 patient charts were investigated from a single center; however, the results would be expected to vary across regions with different population demographics. Another limitation is the operation capacity of endoscopy facilities. During the pandemic, different facilities operated at varying capacities, given their healthcare facility protocols. This aspect also prevents the results of this study from being generalized.

The study supports that colonoscopy rates between whites and African Americans significantly differ, probably because of unequal access to these procedures. Solutions should be aimed at addressing this disparity. As defined in a study by Levesque et al. on patient-centered access to healthcare, access is an opportunity to obtain appropriate healthcare services in situations of perceived need for care [13]. The authors describe the five dimensions of accessibility of services as approachability, acceptability, availability and accommodation, affordability, and appropriateness, which are explained below.

Approachability

Approachability is the ability of people with health needs to identify that a form of service exists, can be obtained, and can influence their health [13]. Missed appointments can be prevented by scheduling patients for their one-year colonoscopy on the same day as their resection; placing follow-up telephone calls and leaving voicemails if patients are unavailable; and ensuring that, before discharge, patients are given a contact number for rescheduling.

Acceptability

Acceptability relates to social factors influencing people’s acceptance of aspects of the service [13]. Sly et al. have reported that African Americans who do not adhere to screening guidelines may not obtain their colonoscopies because they do not know what to expect, thus causing fear and anxiety [3]. Proper education regarding what a colonoscopy entails and its importance to patients before discharge is valuable; moreover, additional follow-up phone calls, letters, or emails reminding patients about this procedure and its importance several days or weeks before the due date would help ensure patient attendance.

Availability and accommodation

Availability and accommodation are major issues because most African Americans live an average of four miles from the hospital where the procedures occur. Having satellite facilities near areas where colonoscopy rates within the population are low could help increase appointment availability, thus allowing more patients to schedule at times that suit them.

Affordability

Affordability reflects the economic ability of people to spend resources and time to use appropriate services [13]. Before discharging patients after their resections, healthcare providers must be transparent regarding the cost of the colonoscopy or, if that information is not available at that time, must inform the patients after the cost becomes available. Providers should also guide patients or provide resources on receiving the appropriate healthcare coverage months in advance before their procedure.

Appropriateness

Appropriateness is the fit between services and the client's need, its timeliness, the amount of care spent in assessing health problems, determining the correct treatment, and the technical and interpersonal quality of the services provided [13]. This dimension of accessibility requires healthcare providers to build a good rapport with their patients, be transparent regarding patient health status, and explain the importance of the treatments that they recommend. Explaining the need for a follow-up colonoscopy one year in advance, even before patients undergo colon tumor resection, would give patients time to process that information and ask questions that could ease their anxieties about the next steps. Moreover, during the pandemic, when resources are more limited than before, these five dimensions of accessibility of services are all the more crucial.

## Conclusions

Before the pandemic, many factors caused unequal access to colonoscopies in patients with colorectal cancer after tumor resection among African Americans. These factors include the distance to the hospital and a lack of information regarding the importance of surveillance colonoscopies after colon tumor resection. The COVID-19 pandemic caused a substantial strain in the performance of colonoscopies, limiting the number of procedures that could be performed in a day. This limitation has exacerbated the already major issue of low colonoscopy rates among African Americans, who belong to the race with the highest risk of developing colorectal cancer. Using the five dimensions of accessibility, healthcare providers can help increase the rates at which African Americans obtain their surveillance colonoscopies one year after colonic tumor resection.
